# Urate Handling in the Human Body

**DOI:** 10.1007/s11926-016-0587-7

**Published:** 2016-04-22

**Authors:** David Hyndman, Sha Liu, Jeffrey N. Miner

**Affiliations:** Ardea Biosciences, Inc., Biology Department, 9390 Towne Centre Drive, San Diego, CA 92121 USA

**Keywords:** Gout, Urate, Uric acid, Excretion, FEUA

## Abstract

Elevated serum urate concentration is the primary cause of gout. Understanding the processes that affect serum urate concentration is important for understanding the etiology of gout and thereby understanding treatment. Urate handing in the human body is a complex system including three major processes: production, renal elimination, and intestinal elimination. A change in any one of these can affect both the steady-state serum urate concentration as well as other urate processes. The remarkable complexity underlying urate regulation and its maintenance at high levels in humans suggests that this molecule could potentially play an interesting role other than as a mere waste product to be eliminated as rapidly as possible.

## Introduction

Urate is produced during the metabolism of endogenous (typically DNA and RNA) and exogenous (food-derived) purines. Once produced, urate cannot be further metabolized by human cells and so must be eliminated by either renal or extra-renal elimination routes (primarily via the intestine and the intestinal flora). The balance of production and elimination determines the concentration of urate in the circulation. Urate, with a pKa of 5.3 [1], is also found in its deprotonated form uric acid; however, uric acid represents only ~1 % of the total urate in blood because of the pH. In urine, more of the urate is unprotonated uric acid due to the lower pH found in urine but at any urine pH above 5.3, more than half the molecule will be in the form of urate.

Elevated serum urate (sUA) is the primary cause of gout, an inflammatory arthritis induced by monosodium urate crystals. Hyperuricemia is defined as sUA concentrations greater than 6.8 mg/dl, which is the in vitro solubility limit of monosodium urate. Gout occurs in patients with sUA above 6.8 mg/dl and gout prevalence increases as sUA rises above the 6.8 mg/dL threshold [2]. International guidelines recommend lowering sUA levels to a target range of <6 mg/dL (<360 μmol/L) in all gout case scenarios and below <5 mg/dl (<300 μmol/L) in those with greater disease severity and urate burden, such as those with tophi [3, 4]. Therefore, clarity on the interplay between factors that affect serum urate handling is a key component to understanding and treating gout.

### Production

As noted above, endogenous production of urate derives from the normal cellular metabolism (turnover) of purines such as DNA, RNA, and ATP. The other source of substrates for urate production is dietary purines that are metabolized to urate in the intestine [5–8]. Therefore, the amount of purines in diet can affect urate production, though a significant reduction in dietary purines is required to have clinically relevant decreases in sUA [9–11]. Other factors that appear to impact production are consumption of fructose and beer [12]. As a result of environmental and physiological changes, sUA levels can vary significantly from day to day.

### Elimination

Elimination of urate occurs via two routes: renal elimination and extra-renal elimination. Urate elimination is a dynamic process mediated by multiple specific import and export transporters in the renal proximal tubule, salivary glands and the intestinal mucosa [13]. The amount of urate excreted via these elimination routes can be quantified as clearance in milliliters per minute of serum (or blood) in the circulation that is cleared. Thus, the total clearance of urate is the sum of the renal clearance and the extra-renal clearance. The total rate of urate removed per minute is the product of the total clearance and the sUA concentration. So, at steady state, for a given rate of production, sUA will settle at a point at which total elimination is equal to production. These relationships form the basis for understanding the dynamics of urate concentration in the serum.

### Renal Elimination

The kidney is typically responsible for approximately 60–65 % of daily urate elimination [14]. Urate in blood is freely filtered in the kidney by the glomerulus. The filtered urate is subjected to significant reabsorption in the proximal tubule. In addition, secretion of urate also occurs. Both these processes are carried out by a series of membrane transporters described later. Of the filtered urate, only 3–10 % is eventually excreted in the urine with the majority reabsorbed in the proximal tubule. Fractional excretion of urate (FEUA) is a description of the net urate reabsorption efficiency of the kidney. FEUA is defined as the percent of filtered urate that is ultimately excreted.

$$ \mathsf{FEUA} = \mathsf{excreted}\ \mathsf{urate}/\mathsf{filtered}\ \mathsf{urate} $$

FEUA can be estimated using serum and urine concentrations of urate and creatinine using the following formula which assumes that creatinine clearance is the same as GFR.

$$ \mathrm{FEUA} = \mathrm{u}\mathrm{U}\mathrm{A}/\mathrm{u}\mathrm{C}\mathrm{r}\ *\ \mathrm{s}\mathrm{C}\mathrm{r}/\mathrm{s}\mathrm{U}\mathrm{A} $$

where *uUA* is the urinary urate concentration, *uCR* is the urinary creatinine concentration, *sCR* is the serum creatinine concentration, and *sUA* is the serum urate concentration.

**Fig. 1:**
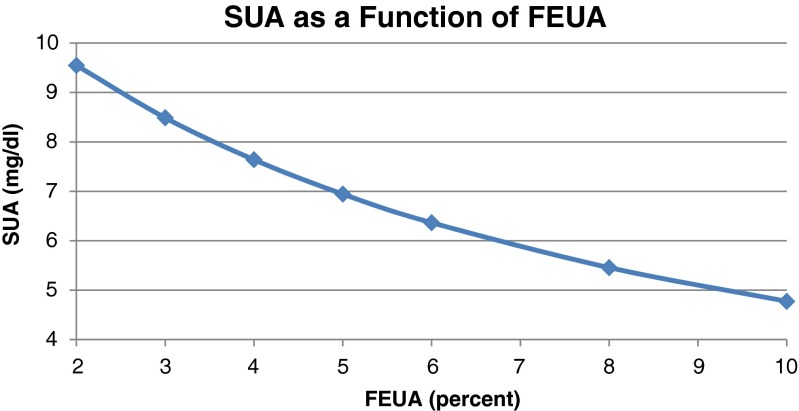
Holding intestinal clearance constant at 6 ml/min, production constant at 1100 mg/day, and GFR constant at 100 mL/min, sUA is calculated as production divided by total clearance (extra-renal plus renal clearance)

While hyperuricemia can be caused by overproduction of urate and decreased intestinal excretion of urate, decreased renal excretion or low FEUA represents a major contributor to hyperuricemia. Healthy subjects have an average FEUA in the range of 6–8 %, whereas gout patients generally have average FEUA of 3–5 %. As seen in Fig. 1, keeping production, GFR, and extra-renal clearance constant, sUA is a function of FEUA.

After filtration by the glomerulus, the urate passes into the proximal tubule where a large portion of the filtered urate is reabsorbed; a smaller portion of urate is secreted as well. However, the degree and location of tubular secretion are a subject of controversy. For many years, the accepted model of renal handling of urate, known as the four-component model, was diligently memorized by students in the field. This model was composed of the following four steps: glomerular filtration, almost complete reabsorption, significant secretion, and then subsequent reabsorption of the secreted urate [15]. This model was based on an incorrect assumption regarding the effect of pyrazinamide and low-dose aspirin on urate transporters in the kidney. It was assumed that these drugs caused an inhibition of secretory transporters and much of the research done for many years after that was designed and interpreted based on those assumptions. However, in 1996, using human kidney brush border vesicles, it was observed that pyrazinoic acid (PZA), a metabolite of pyrazinamide, stimulates uptake of urate [16]. Later, after the cloning and expression of the kidney urate transporter, URAT1, it was found that PZA and salicylic acid both trans-stimulate uptake of urate by URAT1, which neatly explains their activity as stimulators of reabsorption rather than inhibitors of secretion [17]. There have been no reports of inhibition of any secretory transporters by these agents. With this knowledge, many publications that were designed to understand the contributions of reabsorption and secretion can be reexamined in light of this new perspective [15, 18].

Our current view is that, after glomerular filtration, 90–97 % of urate is reabsorbed in the proximal tubule. Tubular secretion of urate does occur; however, it is not yet clear if the secretion happens concomitantly with reabsorption and/or if there is post-reabsorptive secretion within the tubule.

Given the ~180 l of water cycled through the kidney each day together with the rapid cycle of urate filtration, reabsorption and secretion, any given molecule of urate may pass through the kidney multiple times a day before being excreted. This is accomplished via an array of renal transporters driving both reabsorption and secretion of urate.

### Reabsorption of Urate in the Kidney

**Fig. 2:**
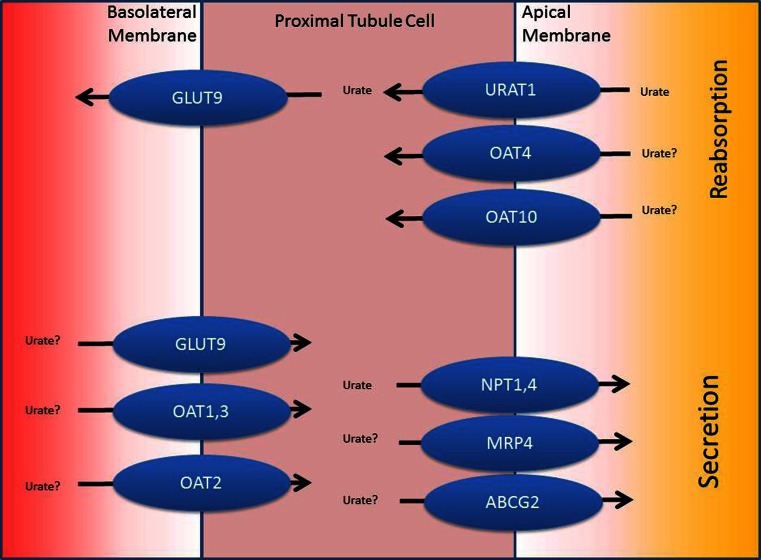
Urate transporters in the kidney—a representative proximal tubule cell is shown with the relevant secretory and resorptive transporters localized to either the basolateral or apical membranes. The *arrows* denote the direction of transport for substrates. The *question marks* for urate and selected transporters denote that questions surround the role of these proteins in urate handling in vivo

No method is available to measure renal urate reabsorption directly. However, because urine urate excretion is less than 10 % of the filtered urate load, there is no question that reabsorption represents a significant component of urate handling by the kidney. Various transporters that play a role in reabsorption have been identified and are shown in Fig. 2.

### Reabsorption Transporters

#### URAT1

URAT1 (*SLC22A12*) has been identified as one of the two most important transporters for urate reabsorption from the apical (luminal) side of the proximal tubule [17]. URAT1 is a typical 12-transmembrane domain protein capable of transporting urate in vitro. Its importance in renal urate reabsorption is confirmed by the observation that individuals who are deficient in functional URAT1 have FEUA of 40–100 % and extremely low serum urate levels [19]. GWAS studies have demonstrated an important role for URAT1 in both hyperuricemia and gout [20]. Additionally, URAT1 is the target for a number of urate lowering therapies (ULTs) that decrease reabsorption of urate. Indeed, all known drugs capable of increasing FEUA (benzbromarone, probenecid, losartan, and lesinurad) inhibit URAT1. Conversely, there are compounds that raise urate levels by increasing the activity of URAT1 and thereby decreasing FEUA. These include both endogenous compounds (lactate and nicotinate) [17], as well as drugs (pyrazinamide and aspirin) [21–23].

#### GLUT9

Glucose transporter 9 (GLUT9, *SLC2A9*), also referred to as URATv1, is present on the basolateral side of proximal tubule cells of the kidney and is the other transporter fundamental to the reabsorption of urate. GLUT9 is a facilitative transporter of urate shown to be strongly linked to both hyperuricemia and gout in GWAS studies [24–28, 29•]. Several reports of subjects with homozygous inactivating GLUT9 mutations demonstrate that these subjects have no net reabsorption of urate presenting with FEUA’s of 100 % or greater. This indicates that it is likely that GLUT9 is the only transporter responsible for the efflux of reabsorbed urate from the interior of the proximal tubule cells back to the blood. GLUT9 actually is present in two different splice variants. The variant SLC2A9-L or long isoform is present on the basolateral side and is responsible for the urate export stage of reabsorption. SLC2A9-S or (short isoform) is expressed in the kidney; however, its function is currently under investigation [30, 31].

#### OAT4

Organic anion transporter 4 (OAT4; *SLC22A11*) is capable of transporting organic anions, including hormones and various drugs in vitro and is expressed on the apical membrane of the proximal tubule. OAT4 also transports urate and shares sequence similarity to URAT1 [32]. OAT4 is associated with hyperuricemia and gout in many genetic studies [25–28]. Sakiyama et al. reported that patients with the OAT4 variant associated with gout and hyperuricemia exhibited inefficient renal excretion, highlighting the role of OAT4 in renal urate handling [33]. OAT4-mediated urate transport has been reported, although the activity is very low compared to that of URAT1 [34] (unpublished).

#### OAT10

OAT10 (*SLC22A13*) is also present on the apical side of the kidney proximal tubule and has been shown to transport urate with low affinity in vitro. Similar to URAT1, the transport of urate by OAT10 is stimulated by exchange of lactate, pyrazinoate, and nicotinate [35]. However, GWAS studies have not suggested a connection between OAT10 and either gout or hyperuricemia [36•].

### Tubular Secretion

Evidence of tubular secretion of urate was first reported in 1950 in a case study of a subject with hypouricemia that had a defect in reabsorption of urate [37]. Urate clearance was greater than that of inulin, indicating that more urate was being excreted than was being filtered. This can only occur when tubular secretion is present.

Further evidence of tubular secretion of urate was reported by Gutman, Yu, and Berger, who infused patients with intravenous urate to produce very high sUA concentrations [38]. These authors described FEUA values greater than 100 % in these subjects, also consistent with the existence of tubular secretion. More recently, subjects with deactivating mutations of GLUT9 were shown to have, in many cases, FEUA values greater than 100 %, once again indicating urate tubular secretion [39]. Finally, the wealth of genetic data (described below) implicating renal secretory transporters in hyperuricemia and the risk of gout cements tubular secretion’s role in urate regulation.

The extent to which urate secretion is concomitant with, versus occurs after reabsorption, remains unclear. Publications describing the effect of pyrazinamide on FEUA demonstrated that when subjects are given a high dose of pyrazinamide, the average FEUA is approximately 0.5 %. Because the only known effect of pyrazinamide on urate handling is to increase reabsorption by URAT1, this indicates that 0.5 % is the upper limit of post-reabsorptive secretion [15]. However, data from various studies including our own indicate that when sUA is decreased, the FEUA decreases as well but importantly levels off at an FEUA of approximately 2 % [23, 40, 41] (Liu et al., manuscript in preparation), which indicates that the total contribution of secretion is somewhat higher than the contribution of post-reabsorptive secretion. This leads to the conclusion that a significant component of secretion occurs concomitantly with reabsorption.

### Tubular Secretion Transporters

Several potential renal secretory transporter candidates have been identified. These can be divided into basolateral transporters, which would transport urate from the interstitial fluid into proximal tubule cells, and apical transporters, which would transport urate from proximal tubule cells into the lumen of the proximal tubule.

ABCG2/BCRP (*ABCG2*), NPT1 (*SLC17A1*), NPT4 (*SLC17A3*), and MRP4 (*ABCC4*) have all been localized on the apical side of the proximal tubule and have all been shown to transport urate in vitro [42–44]. GWAS studies show that ABCG2, NPT1, and NPT4 are associated with hyperuricemia and gout [20, 27, 28, 45]. MRP4 is not associated in these studies, and since no mutations in MRP4 have been found that affect the risk of gout or hyperuricemia, its importance remains unclear. Individual contributions of NPT1 and NPT4 to tubular secretion have been postulated, but their relative roles in the kidney remain incompletely defined. As for ABCG2, the story may be more complicated. ABCG2 was first hypothesized to be functional in renal proximal tubule cells [46]. One study found much higher expression in intestine relative to kidney [47]. Consistent with intestinal expression, the clinical urate excretion phenotype of ABCG2 variants is better explained by an effect on intestinal excretion rather than an effect of urate handling in the kidney, a point discussed more fully later. Since ABCG2’s function in the kidney is unclear, NPT4 and NPT1 remain the apical secretory transporters expressed in the renal apical membrane with the most support for a role in the renal secretion of urate.

Which transporters are involved in tubular secretion on the basolateral side of the proximal tubule is a complex question. If, as is believed, much of the secretion occurs in the same cells of the proximal tubule as reabsorption (concomitant secretion), then much of what is secreted could simply be the urate that was previously reabsorbed. But, because GLUT9-deficient subjects have net secretion of urate, this is consistent with import of urate from the interstitium through the basolateral membrane of the tubule. OAT1, OAT2, and OAT3 have been considered the primary candidates for this because they can transport urate in vitro [48], though there is no supporting genetic data for a role for these transporters. Finally, although GLUT9 is clearly a component of the urate reabsorption system functioning as an *exporter* out of the cell into the interstitium as described earlier, it may also function as an *importer* with a role in secretion. In vitro, GLUT9 is capable of *importing* as well as exporting urate [49], consistent with its facilitative transport mechanism. It could have a role in the movement of urate from the interstium across the basolateral membrane into the proximal tubule cell as part of the tubular secretion machinery. However, because patients with GLUT9 mutations have evidence for continued secretion, then other transporters are likely involved.

### Fractional Excretion of Urate as a Function of sUA

**Fig. 3:**
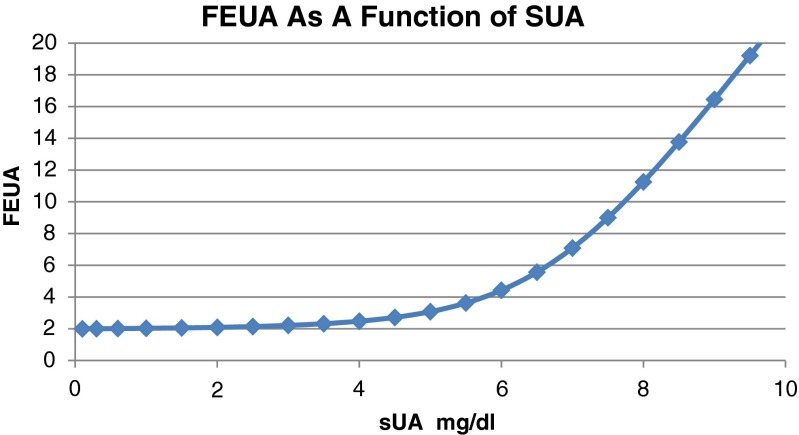
Representation of our view on the effects of changing sUA on FEUA. This is based on our own clinical analysis as well as data from several publications that have reported that FEUA increases as sUA is increased by purine loading or urate infusion. Also, FEUA has been seen to decrease when sUA is decreased by xanthine oxidase inhibitors or low purine diets but does not go below FEUA of 2 % and apparently levels off as sUA approaches zero [9, 18, 23, 40, 50–52]

As mentioned earlier, sUA is determined in part by FEUA. Conversely, FEUA can change as a result of changes in sUA. Several studies have assessed FEUA for the same subjects before and after sUA was modified by means not directly affecting the kidney [9, 18, 23, 40, 50–52]. These include administration of xanthine oxidase inhibitors, purines, or infusion of urate itself. The results from a wide variety of studies indicate that when sUA is increased in a renal-independent fashion, FEUA increases and similarly when sUA decreases, FEUA decreases, as shown in Fig. 3. This dynamic response to sUA levels is likely an inherent property of the transporters found in the kidney. Interestingly, when FEUA is very low at baseline (as is typically true of gout patients), the FEUA does not decrease as much when sUA decreases and the FEUA does not continue to decrease to zero as sUA drops. Instead, FEUA appears to level off at a specific nonzero point (approximately 2 % FEUA). Our own data has corroborated this understanding (manuscript in preparation). While we have not pushed sUA levels below 2 mg/dl in our clinical studies, we have detected the point at which FEUA levels off. The fact that FEUA does not go to zero under these conditions is consistent with the presence of tubular secretion of urate. In addition, we have developed a mathematical model of the proximal tubule to calculate FEUA based on principles of transporter kinetics for reabsorption and secretion that fits these observations.

### Agents Affecting Renal Handling

Pyrazinamide, a drug used to treat tuberculosis, significantly increases serum urate levels [22]. Pyrazinoic acid (PZA), a metabolite of pyrazinamide, stimulates URAT1 activity, which decreases renal urate clearance and increases sUA levels [16, 17]. Low-dose aspirin also causes a decrease in renal clearance of urate. Salicylic acid, the active metabolite of aspirin, stimulates URAT activity [53].

#### Uricosuric Drugs

FDA-approved drugs for the treatment of hyperuricemia and gout are known to inhibit URAT1 and include probenecid and lesinurad (Zurampic). Probenecid is an older medication that is uncommonly used in gout due to difficult dosing schedule and prevalent drug–drug interactions. Lesinurad is a recently FDA-approved selective urate resorption inhibitor (SURI) for the treatment of hyperuricemia associated with gout, capable of lowering urate in combination with a xanthine oxidase inhibitor. Lesinurad was also recently approved in Europe for the treatment of gout. Another URAT1 inhibitor, benzbromarone, is approved for use in Japan but is only available on a named patient basis in Europe and is not approved in the USA due to idiosyncratic liver toxicity. Losartan, an antihypertensive agent that has the additional property of lowering urate level, likely has this effect through inhibition of URAT1 [17].

#### Diuretics

Long-term treatment with either thiazide or loop diuretics causes a lowering of the renal clearance of urate resulting in an increase in serum urate and the risk of gout [54]. These agents may affect urate levels by multiple mechanisms, and a number of hypotheses have been developed with reasonable supporting data. Hypovolemia resulting from diuretic use may be an important effector of diuretic-induced hyperuricemia. This is demonstrated by the fact that salt restriction, which also causes hypovolemia, produces hyperuricemia that is reversed by salt loading [55, 56].

Loop and thiazide diuretics produce an increase in angiotensin II. Angiotensin II increases have been shown to decrease the FEUA resulting in increased sUA [57]. Mechanistically speaking, decreases in FEUA due to angiotensin II may be linked to enhanced URAT1 activity. Increases in angiotensin II cause increased expression of NHE3, a sodium-hydrogen exchanger, among others [58]. NHE3 absorbs sodium and exports hydrogen ions into the lumen of the nephron resulting in a decrease in pH. In vitro URAT1 is more active at lower pH when expressed in human cells (our unpublished data), so increased activity of NHE3 could result in increased URAT1 activity. Further evidence that increased URAT1 activity is the mechanism of diuretic-induced hyperuricemia is the report that diuretics also reduce the clearance of the allopurinol metabolite oxypurinol [59, 60]. Oxypurinol is reabsorbed in the kidney by URAT1 just as urate is [61]. Therefore, it is possible that the decrease in renal clearance of urate due to diuretic use is via an increase in URAT1 activity. However, a genetic association between URAT1 and diuretic induced hyperuricemia has not been found in either published study of this phenomenon.

Other publications suggest the potential involvement of OAT4 in diuretic-induced hyperuricemia. Evidence exists for direct stimulation of OAT4 by hydrochlorothiazide, a thiazide diuretic, and torsemide, a loop diuretic [34, 62], suggesting that this mechanism might be a potential contributor to diuretic-induced hyperuricemia. However, no additional reports of this in vitro phenomenon have surfaced to suggest that all thiazide or loop diuretics would have this same effect. There is conflicting genetic data on OAT4 and diuretic-induced hyperuricemia. One study reported an association [63], while another study did not [64]. Testing selective OAT4 inhibitors in clinical trials or detecting nonsynonymous variants in OAT4 with phenotypic effects are two potential approaches to clarify the role of OAT4 in diuretic-induced hyperuricemia.

#### SGLT2 Inhibitors

Sodium-dependent glucose transporters are responsible for the active transport of glucose across the proximal tubular membrane. Inhibitors of these transporters prevent renal glucose reabsorption and decrease serum glucose levels significantly. These agents as a class also lower uric acid levels by increasing urinary urate clearance. This effect may be due to the action of glucose on GLUT9-mediated urate transport, though the specific mechanism is unclear [30, 65].

### Intestinal Elimination

Based on published experiments calculating production [14], the average person excretes approximately 65 % of their daily urate production via the kidney and the remainder via extra-renal elimination. However, some gout patients have been found to excrete only 40 % via the kidney. Therefore, in some subjects, such as those with renal failure, the relative role of extra-renal elimination may be greater.

As in the kidney, urate transporters are likely involved in urate handling in the intestine. Urate enters the intestine either by secretion from the bloodstream, or as a component of bile, saliva, or peptic juices. Once in the intestine, urate can be reabsorbed as evidenced by the fact that Sevelamer, a nonabsorbable, phosphate-binding polymer that also binds urate and can lower serum urate levels. That which is not reabsorbed is degraded by the uricase activity found in the intestinal microbiome resulting in CO2 or allantoin [66]. Almost no urate is found in feces under normal conditions because of these mechanisms [66].

#### ABCG2

ABCG2, also called BCRP, was first identified as a transporter of xenobiotics and was associated with multi-drug resistance to chemotherapeutic drugs. In 2008, ABCG2 was identified in a GWAS study [45] as being associated with serum urate levels, and in 2009, it was found to transport urate in vitro [46]. Genetic validation came when an ABCG2 variant (Q141K) associated with elevated serum urate and was found to have less urate transport activity, consistent with a role in excretion of urate [67].

ABCG2 is present in both kidney and intestine, but expression in kidney is weak, whereas expression in the small intestine is very strong [68]. Multiple lines of evidence exist supporting its role in secreting urate into the intestine. Ichida et al. [67] reported that the ABCG2 risk allele was associated with hyperuricemia in which renal urate excretion *is increased*. This is consistent with a direct effect on intestinal elimination and an indirect effect on the kidney. Similarly, Dalbeth et al. reported that subjects with the Q141K risk variant have not only higher SUA but also slightly *higher* FEUA [69•]. More recently, Matsuo et.al [70] also reported that the ABCG2 variant responsible for hyperuricemia *increased* FEUA. In contrast, Kottgen et.al. [36•] reported that FEUA is lower in those with the ABCG2 variants associated with hyperuricemia. The Kottgen report is the outlier in this case, and it is difficult to reconcile this result with the others. One explanation for why an increase in FEUA might be observed in patients with ABCG2 dysfunction is that lack of intestinal secretion raises sUA which in turn can indirectly increase FEUA, as mentioned earlier.

One other intestinal transporter that may play a role is NPT5 (*SLC17A4*). This transporter is an anion exporter with homology to sodium-phosphate cotransporters and is specifically expressed on the intestinal brush border membrane. It can transport urate *in vitro* [71]. Furthermore, genome-wide association studies have identified the cluster that includes NPT5 as being associated with circulating urate concentration [72], an important potential hint as to its role in gout.

## Conclusions

Urate handling is a complex, dynamic balance between three major processes: production, renal elimination, and intestinal elimination. A change in any one of these can affect both the steady-state serum urate concentration as well as other urate processes. The remarkable complexity underlying urate regulation and its maintenance at high levels in humans suggests that this molecule could potentially play an interesting role other than as a mere waste product to be eliminated as rapidly as possible.
